# Usability and Technology Acceptance of a Wearable Monitoring System Among Patients with Cardiovascular Diseases: A Cross-Sectional Study

**DOI:** 10.3390/medicina62081607

**Published:** 2026-08-21

**Authors:** Marian Morenci, Diana Carina Iovanovici, Carmen Delia Cseppento Nistor, Sebastian Tirla, Anett Jolán Karetka, Rareș Gheorghe Mihuț, Akos Tiboldi, Lisa Lichtenegger, Laura Ioana Bondar, Tünde Jurca

**Affiliations:** 1 Doctoral School of Biomedical Sciences, Faculty of Medicine and Pharmacy, University of Oradea, 410087 Oradea, Romania; morenci.marian@student.uoradea.ro (M.M.); tirla.sebastian@student.uoradea.ro (S.T.); balasko.anettjolan@student.uoradea.ro (A.J.K.); mihut.raresgheorghe@student.uoradea.ro (R.G.M.); tjurca@uoradea.ro (T.J.); 2 Timișoara Institute of Cardiovascular Diseases, 300310 Timișoara, Romania; diana_iovanovici@yahoo.com; 3 Department of Psycho-Neuroscience and Recovery, Faculty of Medicine and Pharmacy, University of Oradea, 410073 Oradea, Romania; 4 Department of Pharmacy, Faculty of Medicine and Pharmacy, University of Oradea, 410073 Oradea, Romania; 5 Ludwig Boltzmann Institute for Traumatology, The Research Center in Cooperation with AUVA, 1200 Vienna, Austrialisa.lichtenegger@lbg.ac.at (L.L.); 6 Ludwig Boltzmann Institute for Digital Health and Patient Safety, 1090 Vienna, Austria; 7 Department of Anaesthesia, Intensive Care Medicine and Pain Medicine, Clinical Division of General Anaesthesia and Intensive Care Medicine, Medical University of Vienna, 1090 Vienna, Austria; 8 Department of Biology and Life Sciences, Faculty of Medicine, “Vasile Goldiș” Western University of Arad, 310025 Arad, Romania

**Keywords:** cardiovascular diseases, digital health, patient acceptance, remote patient monitoring, System Usability Scale, Technology Acceptance Model, wearable electronic devices

## Abstract

*Background and Objectives:* Wearable health technologies have become increasingly important in the remote management of cardiovascular diseases (CVDs); however, their successful implementation depends not only on technical performance but also on patient usability and technology acceptance. This study aimed to evaluate the perceived usability and technology acceptance of a wearable monitoring system among patients with CVDs and to investigate whether demographic and clinical characteristics influenced these outcomes. *Materials and Methods:* A cross-sectional observational study was conducted among 60 patients with CVDs enrolled in a six-month wearable monitoring program at the Medical Rehabilitation Outpatient Clinic of the Avram Iancu Clinical Hospital, Oradea, Romania. Participants used a Garmin vívosmart^®^ 5 fitness tracker to continuously monitor physiological and behavioral parameters. At the end of the monitoring period, perceived ease of use (PEU) was assessed using the System Usability Scale (SUS), while technology acceptance was evaluated using a Technology Acceptance Model (TAM)-based questionnaire. Descriptive statistics, reliability analysis, Spearman’s rank correlation, and multiple linear regression analyses were performed. *Results:* The wearable monitoring system demonstrated high PEU, with a median SUS score of 82.5 (IQR = 23.1) and a mean score of 75.5 ± 17.6. Overall, 75.0% of participants rated the system as having good or excellent usability. The SUS questionnaire demonstrated acceptable internal consistency (Cronbach’s α = 0.730; McDonald’s ω = 0.741). Technology acceptance was generally favorable, with an overall median TAM-based score of 1.33 (IQR = 0.78). Age and comorbidity burden were not associated with PEU, and no participant characteristics independently predicted SUS scores. Although the overall regression model was not statistically significant, age showed a statistically significant association with the overall TAM-based score (β = 0.328, *p* = 0.037). Significant positive correlations were observed between PU, self-efficacy (SE), and reverse-scored technology anxiety (TA) measure (*p* < 0.001). *Conclusions:* Patients with CVDs reported high PEU and favorable acceptance of wearable monitoring technology. While usability appeared independent of demographic and clinical characteristics, technology acceptance showed an exploratory association with age, although the overall regression model was not statistically significant. These findings support the integration of wearable monitoring systems into routine cardiovascular care and highlight the importance of patient-centered implementation strategies to promote long-term engagement.

## 1. Introduction

Cardiovascular diseases (CVDs) remain the leading cause of morbidity and mortality worldwide, accounting for a substantial proportion of healthcare utilization, hospital admissions, and premature deaths. The growing prevalence of hypertension, heart failure, atrial fibrillation, and other chronic cardiovascular conditions has increased the demand for innovative strategies capable of supporting long-term disease management and reducing the burden on healthcare systems. Advances in digital health technologies have created new opportunities for continuous patient monitoring, early detection of clinical deterioration, and personalized disease management outside traditional clinical settings [1,2,3,4,5].

Wearable health technologies have emerged as an important component of digital cardiovascular care. Modern wearable devices are capable of continuously monitoring physiological parameters such as heart rate, cardiac rhythm, physical activity, blood oxygen saturation, and sleep patterns, providing both patients and healthcare professionals with real-time health information. Previous studies have demonstrated that wearable monitoring may improve symptom recognition, facilitate earlier clinical intervention, promote patient engagement, and support remote management of chronic CVDs [6,7,8,9,10,11]. Consequently, wearable technologies are increasingly being incorporated into telemedicine programs and hybrid models of cardiovascular care.

Despite their technological capabilities, the clinical effectiveness of wearable devices depends largely on patient acceptance and sustained engagement. Even highly accurate monitoring systems may fail to provide clinical benefit if patients perceive them as difficult to use, unreliable, or burdensome during everyday activities. For this reason, human factors such as usability, perceived usefulness (PU), self-efficacy (SE), attitude toward use (AT), technology-related anxiety (TA), and perceived effects on quality of life (QoL) have become increasingly recognized as important determinants of successful implementation of digital health interventions [12,13,14].

Several validated instruments have been developed to evaluate these constructs. The System Usability Scale (SUS) is one of the most widely used questionnaires for assessing perceived ease of use (PEU) of medical technologies because of its simplicity, reliability, and extensive validation across healthcare settings [15,16,17,18]. Similarly, the Technology Acceptance Model (TAM) provides a theoretical framework for understanding users’ acceptance of new technologies, with PU representing one of its core constructs. Extended technology-acceptance frameworks have incorporated additional individual and contextual factors, including SE, TA, and health-related characteristics [19,20,21,22]. Together, these instruments provide complementary information regarding both the practical usability and broader acceptance of wearable monitoring technologies.

Although wearable devices have been extensively investigated in recent years, several important knowledge gaps remain. Most published studies have focused primarily on device accuracy, algorithm performance, or short-term feasibility, whereas fewer investigations have comprehensively evaluated both usability and technology acceptance among patients with established CVDs using validated psychometric instruments. Furthermore, limited evidence is available regarding whether demographic and clinical characteristics, including age, comorbidity burden, residential environment, and employment status, influence patients’ perceptions of wearable technologies. Addressing these gaps is essential for identifying potential barriers to technology acceptance and continued use and optimizing the implementation of wearable monitoring in routine cardiovascular care [23,24].

Therefore, the aim of the present study was to evaluate the PEU and technology acceptance of a wearable monitoring system among patients with CVDs using the SUS and an adapted TAM-based questionnaire. In addition, we investigated whether demographic and clinical characteristics were associated with PEU and technology acceptance. We hypothesized that the wearable monitoring system would demonstrate good PEU and high technology acceptance and that participant characteristics, including age and comorbidity burden, might influence these outcomes.

## 2. Materials and Methods

### 2.1. Study Design

This study employed an observational cross-sectional design nested within a six-month prospective wearable monitoring program to evaluate the PEU and technology acceptance of a wearable monitoring system among patients with CVDs. The study was conducted between January 2025 and February 2026 at the Outpatient Cardiology Clinic of Avram Iancu Clinical Hospital, Oradea, Romania.

A total of 60 consecutive patients who fulfilled the study eligibility criteria were enrolled. At the baseline visit, participants underwent a clinical assessment that included demographic data collection, cardiovascular risk evaluation, medical history, comorbidity assessment, and lifestyle evaluation.

Following the initial assessment, participants received a wearable fitness bracelet as part of the DIGI4Care (Digital Medical Technologies Reshaping the Danube Region Healthcare Systems Throughout the Patient Journey from Prevention to Rehabilitation) project and were instructed to wear the device continuously for six months (August 2025–January 2026). The wearable device continuously monitored health-related parameters, including daily step count, distance traveled, energy expenditure, heart rate, stress level, and sleep duration. Device data were synchronized through a dedicated smartphone application, enabling participants to monitor their health indicators in real time.

At the end of the six-month monitoring period, participants attended a follow-up visit during which PEU and technology acceptance of the wearable monitoring system were assessed using the SUS and a TAM-based questionnaire. Clinical data were linked using unique study identification codes, and all datasets were anonymized prior to statistical analysis to ensure participant confidentiality.

### 2.2. Study Population

Participants were consecutively recruited from the Medical Rehabilitation Outpatient Clinic at the Avram Iancu Clinical Hospital in Oradea, Romania. Eligible participants were adults diagnosed with CVDs who were enrolled in the DIGI4Care project and agreed to participate in the six-month wearable monitoring program.

Participants were eligible for inclusion if they met all of the following criteria:

- Age ≥ 18 years;
- Confirmed diagnosis of a CVD (e.g., hypertension, heart failure, cardiac arrhythmias, or valvular heart disease);
- Ability to use a smartphone to synchronize wearable device data;
- Willingness to wear the provided fitness bracelet throughout the six-month monitoring period;
- Provision of written informed consent.

Participants were excluded if they met any of the following criteria:

- Inability to operate a smartphone or synchronize the wearable device;
- Discontinuation of wearable device use before completion of the monitoring period;
- Incomplete baseline assessment;
- Withdrawal of informed consent during the study.

**Figure 1:**
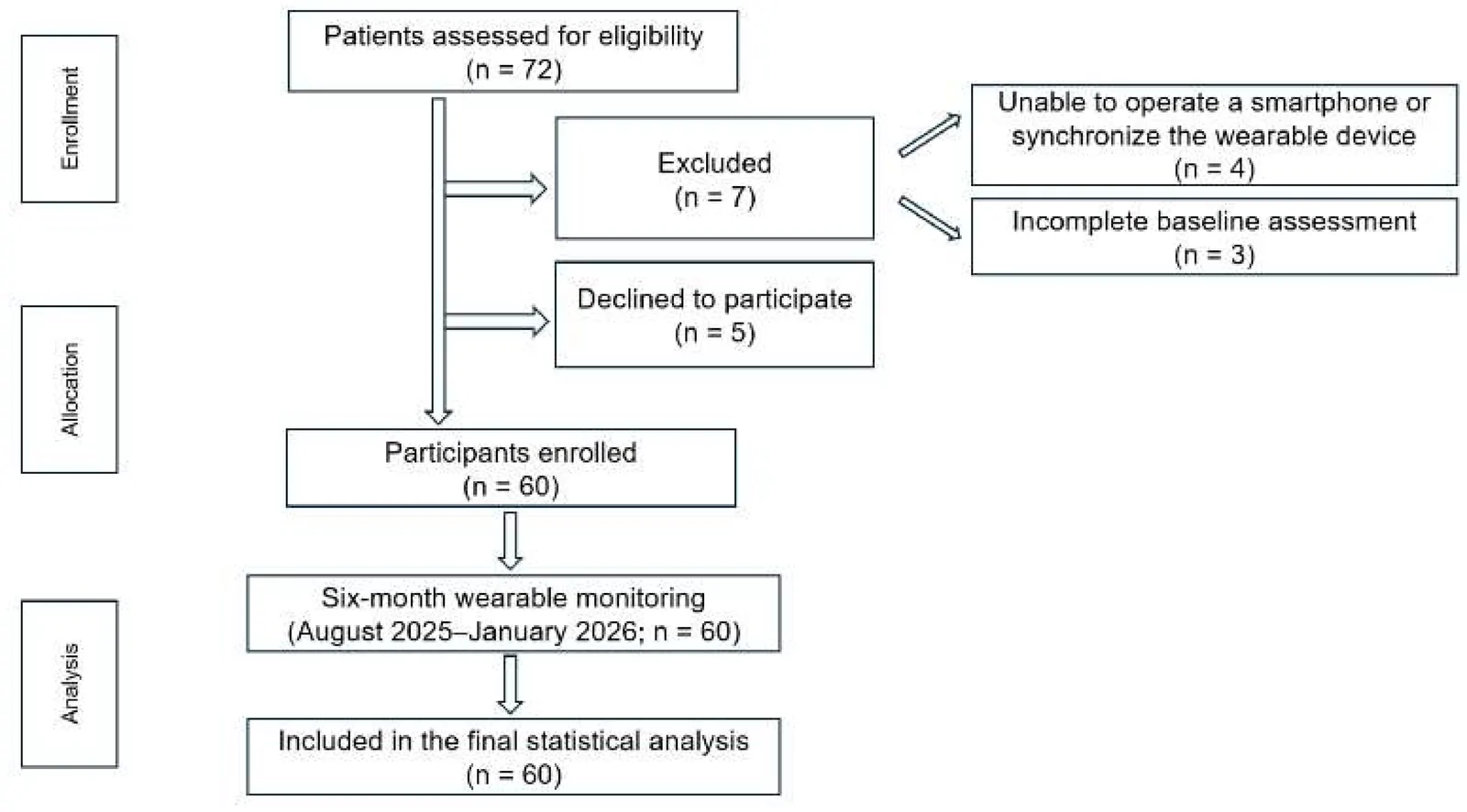
Flowchart of participant recruitment and inclusion in the study.

Of the 72 patients assessed for eligibility, 5 declined to participate and 7 were excluded (4 because they were unable to operate a smartphone or synchronize the wearable device and 3 because of an incomplete baseline assessment), resulting in 60 participants being enrolled. All 60 enrolled participants completed the six-month monitoring period and were included in the final statistical analysis (Figure 1).

Recruitment was intended to obtain a general, clinically heterogeneous cardiovascular cohort for the overall evaluation of wearable-device usability and technology acceptance. The sample was not designed or powered for diagnosis-specific subgroup analyses. In particular, some clinical subgroups were small, including patients with valvular heart disease and heart rhythm disorders (*n* = 5, 8.3% each); therefore, no diagnosis-specific comparisons were performed, and the findings should not be interpreted as evidence of comparable usability or technology acceptance across individual cardiovascular diagnoses.

### 2.3. Wearable Monitoring System and Study Procedures

Participants enrolled in the study received a Garmin vívosmart^®^ 5 fitness tracker (model A04352; Garmin International, Inc., Olathe, KS, USA) as part of the DIGI4Care project. Before initiating the monitoring period, all participants received standardized instructions regarding device use, synchronization procedures, and the functionalities of the accompanying smartphone application.

The Garmin vívosmart^®^ 5 was paired with the Garmin Connect™ mobile application, which enabled automatic synchronization of physiological and behavioral data throughout the study period. Participants were instructed to wear the fitness tracker continuously for six months, removing it only when necessary (e.g., for charging or activities incompatible with device use). Continuous wear was encouraged to maximize data completeness and accurately reflect participants’ daily physiological status and physical activity.

Throughout the monitoring period, the wearable device continuously recorded several health-related parameters, including daily step count, distance traveled, energy expenditure (calories burned), heart rate, stress level, and sleep duration. Data were automatically synchronized with the Garmin Connect™ application, allowing participants to access their health metrics in real time while enabling continuous remote monitoring.

The present analysis was specifically designed to evaluate patient-reported usability and technology acceptance of the wearable monitoring system. Accordingly, longitudinal physiological and behavioral outcomes derived from the wearable device, including daily step count, distance traveled, energy expenditure, heart rate, stress level, and sleep duration, were outside the scope of the present analysis and will be evaluated separately as part of the clinical assessment of the monitoring intervention.

At the completion of the six-month monitoring period, participants attended a follow-up visit during which PEU and technology acceptance of the wearable monitoring system were assessed using the SUS and a TAM-based questionnaire.

### 2.4. Study Variables and Outcome Measures

Demographic and clinical characteristics were collected during the baseline assessment, whereas PEU and technology acceptance were evaluated at the completion of the six-month wearable monitoring period using standardized questionnaires.

#### 2.4.1. Demographic and Clinical Variables

Baseline demographic variables included age, sex, place of residence (urban/rural), and professional status. Clinical characteristics comprised the primary cardiovascular diagnosis, including hypertension, heart failure, valvular heart disease, heart rhythm disorders, diabetes mellitus, and the presence of additional comorbidities. Overall comorbidity burden was quantified using the Charlson Comorbidity Index (CCI), a validated prognostic instrument that estimates disease burden based on the number and severity of chronic medical conditions [25].

Behavioral variables included adherence to wearable device use throughout the monitoring period, classified as continuous use, intermittent use (>75% of the monitoring period), or sporadic use (<75% of the monitoring period). Participants were also asked whether the use of the wearable device had resulted in lifestyle modifications during the monitoring period.

#### 2.4.2. SUS

PEU of the wearable monitoring system was assessed using the SUS, a validated questionnaire developed by Brooke [15,17,26]. The SUS consists of 10 items rated on a five-point Likert scale, ranging from 1 (strongly disagree) to 5 (strongly agree). Responses were scored according to the standard SUS scoring procedure, yielding a total score ranging from 0 to 100, with higher scores indicating better PEU.

For descriptive purposes, SUS scores were interpreted according to established usability categories: poor (<51), acceptable (51.0–67.9), good (68.0–80.2), and excellent (>80.3).

#### 2.4.3. TAM-Based Questionnaire

Technology acceptance was assessed using a 12-item questionnaire adapted from the conceptual framework of the TAM and extended with constructs considered relevant to the wearable-health context [21,27,28,29,30]. The instrument was not the original TAM3 questionnaire described by Venkatesh and Bala and does not reproduce the complete TAM3 construct structure. Rather, it represents an adapted TAM-based questionnaire incorporating technology-acceptance constructs together with contextual health-related factors.

The questionnaire consisted of 12 items evaluating four dimensions relevant to the acceptance of wearable health technologies:

- Perceived Usefulness (PU; 2 items);
- Self-efficacy (SE; 3 items);
- Technology anxiety (TA; 2 items);
- Perceived impact on quality of life (QoL; 5 items).

PU was derived from the PU construct of the classic TAM [31], whereas SE and TA were informed by technology/computer SE and anxiety constructs described in the technology-adoption literature [32]. The QoL domain incorporated contextual health- and quality-of-life-related dimensions conceptually informed by the WHOQOL-BREF framework [33]. The complete questionnaire, including individual item wording, construct allocation, scoring direction, and the theoretical sources informing each construct, is provided in Supplementary Materials File S1.

Each item was rated using a five-point Likert scale. For PU, SE, and QoL, lower scores represented more favorable perceptions. Because the TA items assessed anxiety toward technology, these items were reverse-scored before calculation of the TA domain score and the overall TAM-based score. Domain scores were calculated as the arithmetic mean of the corresponding questionnaire items, and the overall TAM-based score was obtained by averaging the scored questionnaire responses. Lower PU, SE, QoL, and overall TAM-based score therefore reflected more favorable perceptions and greater technology acceptance. The TA score should be interpreted according to its reverse-scored direction.

Because the scoring direction is not uniform across the original questionnaire items, the direction of the relevant domain scores is explicitly stated when interpreting descriptive statistics, correlations, and regression coefficients.

The TAM-based questionnaire was administered together with the SUS at the end of the six-month monitoring period to provide a comprehensive evaluation of participants’ perceptions regarding both the usability and acceptance of the wearable monitoring system.

### 2.5. Statistical Analysis

Statistical analyses were performed using IBM SPSS Statistics version 29.0 (IBM Corp., Armonk, NY, USA). Continuous variables were initially assessed for normality using the Shapiro–Wilk test. Normally distributed variables were expressed as mean ± standard deviation (SD), whereas non-normally distributed variables were summarized as median and interquartile range (IQR). Categorical variables were presented as frequencies and percentages.

The internal consistency of the SUS was evaluated using Cronbach’s alpha (α) and McDonald’s omega (ω) coefficients. A 95% confidence interval (CI) was calculated for Cronbach’s alpha to further assess the reliability of the questionnaire.

Because the SUS and TAM-based questionnaire scores were not normally distributed, Spearman’s rank correlation coefficient (ρ) was used to examine associations between continuous variables. Correlation analyses were performed to investigate the relationships among participant characteristics, PEU, and the domains of the TAM-based questionnaire. Given the exploratory nature of the correlation analyses and the number of pairwise comparisons, the Benjamini–Hochberg false discovery rate (FDR) procedure was applied as a sensitivity analysis to account for multiple testing. The FDR correction was applied to the families of pairwise correlation tests; the two prespecified multivariable regression models were treated as separate exploratory analyses, and their coefficients were therefore not subjected to additional multiplicity adjustment but were interpreted cautiously together with the overall model significance and 95% CIs.

Multiple linear regression analyses were performed to identify independent predictors of SUS score and overall technology acceptance (overall TAM-based score). Separate regression models were constructed using the SUS score and the overall TAM-based score as dependent variables. Independent variables included age, place of residence (urban/rural), professional status, and the CCI. Regression results were reported as unstandardized regression coefficients (B) with 95% CIs, standardized regression coefficients (β), coefficients of determination (R^2^), adjusted R^2^, F statistics, and corresponding *p*-values.

Because the study included a consecutive sample of patients enrolled in the DIGI4Care project, no a priori sample size calculation was performed. Given the relatively small sample size, the regression analyses were considered exploratory, and effect estimates were interpreted together with their 95% CIs to reflect the precision of the estimates.

All statistical tests were two-tailed, and a *p*-value < 0.05 was considered statistically significant. For the FDR sensitivity analysis, an FDR-adjusted *p*-value < 0.05 was considered statistically significant.

### 2.6. Ethical Considerations

The study was conducted in accordance with the principles of the Declaration of Helsinki and was approved by the Institutional Ethics Committee of the University of Oradea under approval no. CEFMF/2/27.06.2024. All participants received information regarding the study objectives, procedures, and data confidentiality measures before enrollment.

Written informed consent was obtained from all participants prior to inclusion in the study. Each participant was assigned a unique identification code, and all collected data were anonymized before statistical analysis to ensure confidentiality and protect participant privacy.

## 3. Results

### 3.1. Participant Characteristics

A total of 60 patients were included in the final analysis. The demographic, clinical, and behavioral characteristics of the study cohort are summarized in Table 1.

The median age of the participants was 59.0 years (IQR = 12.5), with a mean age of 59.0 ± 10.07 years. Approximately one-third of the cohort resided in rural areas (31.7%), and most participants were professionally active (63.3%).

Hypertension was the most prevalent cardiovascular diagnosis, affecting 49 participants (81.7%). Heart failure (New York Heart Association—NYHA class I–II) was present in 7 participants (11.7%), while valvular heart disease and heart rhythm disorders were each identified in 5 participants (8.3%). Diabetes mellitus was reported in 8 participants (13.3%), whereas 54 participants (90.0%) presented with additional comorbidities. The median CCI score was 1.0 (IQR = 1.0), indicating a generally low comorbidity burden.

Most participants reported continuous wearable device use throughout the study period. Continuous device use was reported by 40 participants, whereas 15 participants used the device intermittently for more than 75% of the monitoring period and 5 participants reported sporadic use (<75% of the monitoring period). Thus, 55 of 60 participants (91.7%) reported continuous device use or use for more than 75% of the six-month monitoring period, suggesting a high level of sustained engagement with the monitoring system. Lifestyle modifications attributed to wearable monitoring were reported by 15 participants.

### 3.2. PEU of the Wearable Monitoring System

**Figure 2:**
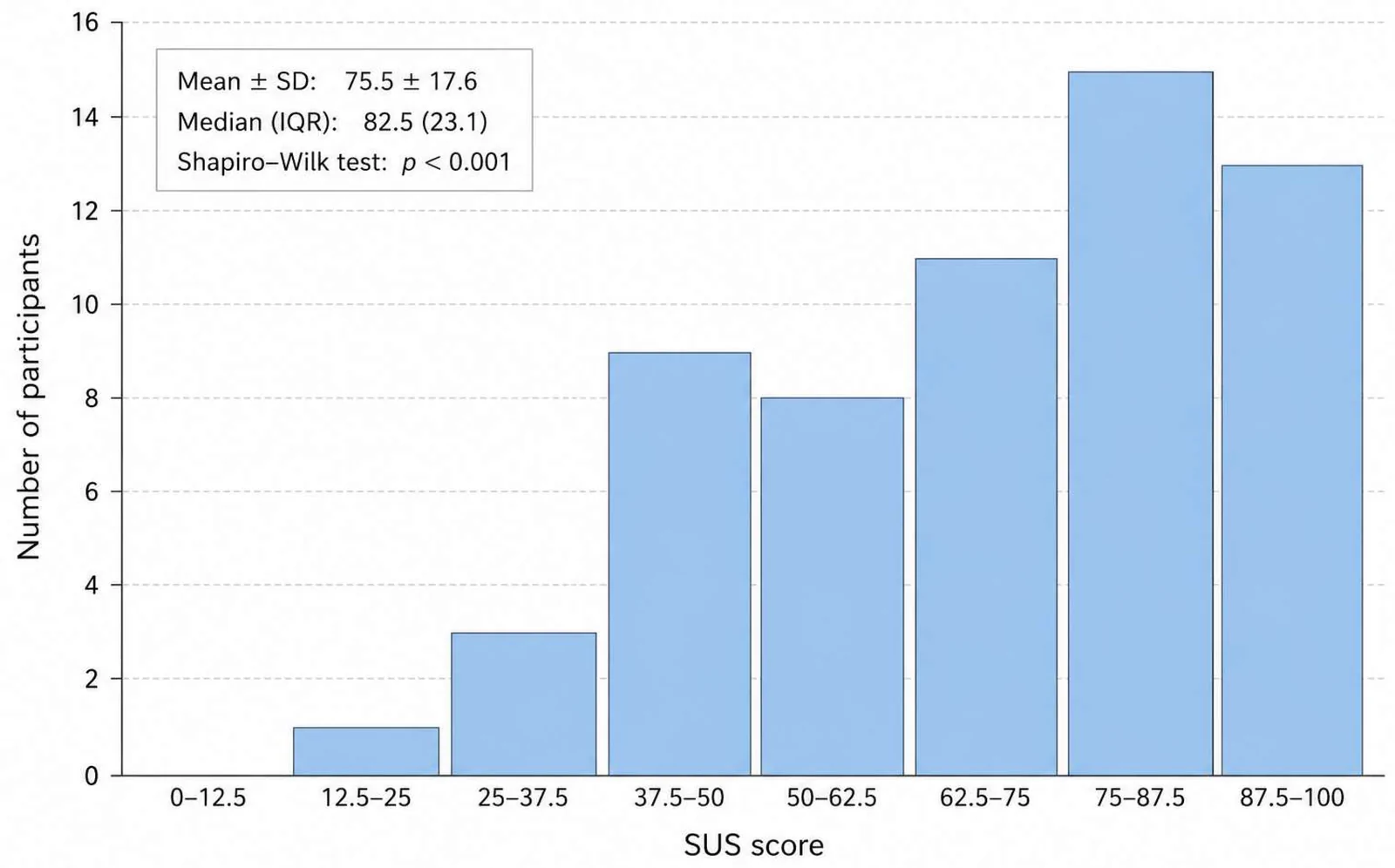
Distribution of SUS scores among study participants.

The distribution of SUS scores deviated significantly from normality according to the Shapiro–Wilk test (*p* < 0.001) (Figure 2). The mean SUS score was 75.5 ± 17.6, whereas the median score was 82.5 (IQR = 23.1).

Based on the established SUS interpretative categories, 35 participants (58.3%) rated the wearable monitoring system as having excellent usability, 10 (16.7%) as good, 7 (11.7%) as acceptable, and 8 (13.3%) as poor (Table 2). Overall, three-quarters of the participants (75.0%) rated the wearable monitoring system as having good or excellent usability.

The SUS questionnaire showed acceptable internal consistency, with a Cronbach’s alpha coefficient of 0.730 (95% CI: 0.614–0.818) and a McDonald’s omega coefficient of 0.741 (Table 3).

### 3.3. Factors Associated with PEU

Spearman’s correlation analysis showed no statistically significant association between age and the SUS score (ρ = 0.106, *p* = 0.422; FDR-adjusted *p* = 0.844). Similarly, no significant correlation was observed between CCI and SUS score (ρ = −0.009, *p* = 0.946; FDR-adjusted *p* = 0.946). These findings remained non-significant after correction for multiple comparisons (Table 4).

A multiple linear regression model was constructed to evaluate whether age, residential environment, professional status, and comorbidity burden predicted SUS scores. The overall model was not statistically significant (F(4,55) = 0.97, *p* = 0.428) and explained 6.6% of the variance in SUS score (R^2^ = 0.066; adjusted R^2^ = 0.002). None of the evaluated variables emerged as significant independent predictors of SUS score (Table 5). Given the CIs around the effect estimates, small positive or negative associations cannot be excluded.

### 3.4. Technology Acceptance of the Wearable Monitoring System

The distribution of TAM-based domain scores deviated significantly from normality according to the Shapiro–Wilk test (all *p* < 0.001). Descriptive statistics for each TAM-based domain are presented in Table 6. For interpretation, lower scores for PU, SE, QoL, and the overall TAM-based score indicate more favorable responses, whereas the TA domain was reverse-scored before analysis. Accordingly, the direction of the TA score should be considered separately when interpreting this domain.

**Figure 3:**
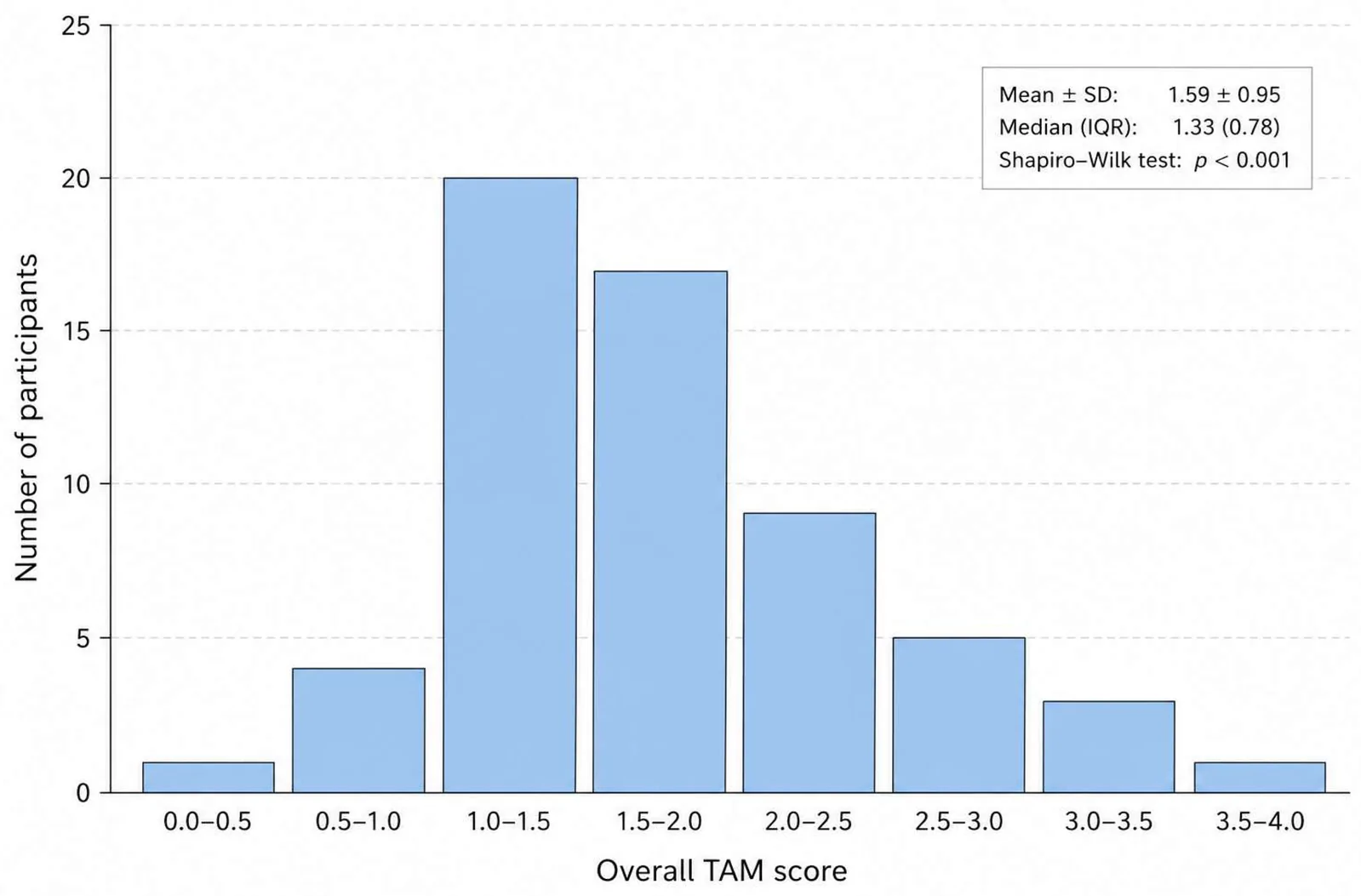
Distribution of the overall TAM-based score among study participants. Lower overall TAM-based score indicates more favorable technology acceptance.

The median scores for PU, SE, and TA were each 1.0, whereas the median quality-of-life score was 1.4. The median overall TAM-based score was 1.33 (IQR = 0.78). Given the scoring direction of the questionnaire, the relatively low PU, SE, QoL, and overall TAM-based score indicates generally favorable responses toward the wearable monitoring system. TA was reverse-scored and should therefore be interpreted according to its corresponding scoring direction. The distribution of the overall TAM-based score is presented in Figure 3.

### 3.5. Correlations Among TAM-Based Domain and SUS Score

Spearman’s rank correlation analysis was performed to evaluate the relationships among the TAM-based domains, QoL, and SUS score. Given the number of pairwise comparisons, the Benjamini–Hochberg false discovery rate (FDR) procedure was applied as a sensitivity analysis to account for multiple testing. The results are presented in Table 7.

Spearman’s correlation analysis identified significant positive correlations between PU and SE (ρ = 0.441, *p* < 0.001), PU and TA (ρ = 0.647, *p* < 0.001), and SE and TA (ρ = 0.491, *p* < 0.001). Because TA was reverse-scored before analysis, the positive correlations involving TA refer to the reverse-scored TA measure and should not be interpreted as indicating that greater PU or SE was associated with greater TA. Rather, the positive coefficients indicate that the corresponding numerical domain scores tended to vary in the same direction according to the applied scoring scheme. All three associations remained statistically significant after FDR correction.

No statistically significant correlations were observed between QoL and the remaining TAM-based domain Likewise, SUS score was not significantly correlated with any TAM-based domain, and these findings remained non-significant after FDR adjustment.

### 3.6. Predictors of Technology Acceptance

A multiple linear regression model was constructed to identify independent predictors of the overall TAM-based score. Age, residential environment, and CCI were included as explanatory variables. As defined by the applied scoring procedure, lower overall TAM-based score indicates more favorable technology acceptance; therefore, positive regression coefficients indicate an increase in the numerical TAM-based score and, consequently, a less favorable acceptance profile.

The overall regression model was not statistically significant (F(3,56) = 2.28, *p* = 0.088), explaining 10.9% of the variance in the overall TAM-based score (R^2^ = 0.109; adjusted R^2^ = 0.061). Age showed a statistically significant positive regression coefficient (B = 0.33, 95% CI: 0.02 to 0.64; β = 0.328, *p* = 0.037), indicating that increasing age was associated with a higher numerical TAM-based score and thus a less favorable technology acceptance profile according to the applied scoring direction. Residential environment and comorbidity burden were not significantly associated with the overall TAM-based score. However, because the overall regression model was not statistically significant, the association observed for age should be considered exploratory and interpreted cautiously (Table 8).

## 4. Discussion

The present study evaluated the PEU and technology acceptance of a wearable monitoring system among patients with CVDs. Overall, the findings partially supported the initial hypothesis. The wearable monitoring system demonstrated favorable PEU, with a median SUS score of 82.5 and 75.0% of participants rating the system as having good or excellent usability. In parallel, TAM-based domain scores indicated a generally favorable acceptance profile, suggesting that participants perceived the wearable monitoring system as acceptable for routine use, consistent with previous reports emphasizing the importance of usability and user acceptance for the successful implementation of digital health technologies [34,35,36].

Contrary to our initial hypothesis, demographic and clinical characteristics were not significantly associated with PEU. Neither age nor comorbidity burden correlated with SUS scores, and none of the evaluated participant characteristics independently predicted PEU. These findings suggest that usability was perceived relatively consistently within the present cohort; however, the small and unevenly represented diagnostic subgroups preclude conclusions regarding whether usability is comparable across specific CVD categories [24,37,38,39,40].

Regarding technology acceptance, the overall regression model was not statistically significant. Nevertheless, age showed a statistically significant regression coefficient, although this finding should be interpreted cautiously because the overall model did not reach statistical significance. Furthermore, PU was positively correlated with SE and the reverse-scored TA measure, whereas QoL was not significantly associated with the other TAM-based domains. These findings suggest that technology acceptance may be related to users’ cognitive perceptions and confidence in using digital technologies. Because the overall regression model was not statistically significant, no independent demographic predictor of technology acceptance could be established in this study [21,22,41,42]. Preliminary findings based on the same cohort of 60 participants were previously presented as a conference abstract at the European Healthcare Management Conference (EHMC) 2026 [43]. The present manuscript reports the complete dataset and provides a substantially expanded analysis of wearable-system usability and technology acceptance.

### 4.1. Interpretation of Wearable Monitoring System Usability

One of the principal findings of the present study was the high PEU of the wearable monitoring system. Participants reported a median SUS score of 82.5, with three-quarters of the study population (75.0%) rating the system as having good or excellent usability. According to the established interpretation of SUS scores, values above 80 indicate excellent usability and are generally associated with a high likelihood of user acceptance and recommendation [6,15,24,38]. These findings suggest that patients generally perceived the wearable monitoring system as usable and acceptable for self-monitoring, supporting its potential integration into routine cardiovascular care.

The observed usability scores are consistent with previous studies evaluating wearable technologies in healthcare. Numerous investigations have reported that wearable monitoring devices are generally perceived as easy to use, particularly when they provide intuitive interfaces, continuous passive monitoring, and minimal user interaction [44,45,46]. In cardiovascular care, high usability has been associated with greater adherence to long-term monitoring, improved patient engagement, and increased confidence in disease self-management [2,47,48]. These study findings therefore support the growing body of evidence indicating that usability represents one of the important factors for successful implementation of wearable health technologies.

An interesting finding of the present study was that PEU was not significantly associated with age or comorbidity burden. Furthermore, none of the evaluated demographic or clinical characteristics independently predicted SUS scores. These results suggest that, once patients become familiar with the wearable monitoring system, PEU may depend more on the quality of the device design than on individual patient characteristics. Similar observations have been reported in studies demonstrating that user-centered design and simplified interaction can substantially reduce age-related barriers to digital health technology adoption [49,50,51,52,53]. Although older adults are frequently considered less willing to adopt digital technologies, previous research has shown that appropriately designed wearable systems can achieve high usability across different age groups [54,55,56].

The favorable usability observed in the present study may also reflect the characteristics of the evaluated monitoring system. Wearable devices capable of automatically collecting physiological data with minimal user input reduce the cognitive and technical demands placed on patients, thereby facilitating long-term engagement. In addition, the relatively high proportion of participants reporting continuous device use throughout the monitoring period may have contributed to the positive usability ratings observed in this study. Previous investigations have similarly demonstrated that sustained interaction with wearable technologies promotes user familiarity, confidence, and satisfaction, ultimately improving the overall user experience [45,57,58,59,60].

Overall, our findings indicate that the evaluated wearable monitoring system achieved a high level of PEU among patients with CVDs, supporting its potential use in routine remote monitoring and digital cardiovascular care. These results reinforce previous evidence that usability constitutes a fundamental prerequisite for the successful implementation of wearable technologies in clinical practice [6,61,62].

### 4.2. Interpretation of Technology Acceptance Findings

Beyond demonstrating high PEU, the present study showed a generally favorable level of technology acceptance among patients with CVDs. Median scores for PU, SE, TA, and the overall TAM-based score indicated that participants were receptive to the wearable monitoring system and experienced relatively few barriers to its use. These findings support the second part of our hypothesis and are consistent with the growing body of evidence suggesting that successful implementation of digital health technologies depends not only on technical performance but also on users’ perceptions of their usefulness and ease of integration into daily life [45,63,64].

The TAM has been widely applied to explain patients’ willingness to adopt digital health technologies, including wearable devices, telemonitoring platforms, and mobile health applications [42,65]. Within TAM and extended technology-acceptance frameworks, PU and users’ confidence in their ability to operate a technology have been identified as important factors associated with technology acceptance and continued use. Previous studies have consistently reported that PU is associated with greater acceptance and continued use of wearable technologies [20,42,66]. The favorable TAM-based score observed in our study therefore suggest that participants recognized the potential value of wearable monitoring in supporting the management of CVD.

An important finding was the positive correlation between PU and SE, indicating that participants who believed the wearable system was beneficial also reported greater confidence in their ability to use it. This observation is consistent with previous applications of the TAM, in which SE has been identified as an important facilitator of digital health adoption [57,67,68]. Previous studies suggest that individuals who feel capable of using health technologies experience fewer practical barriers and greater willingness to integrate these tools into daily healthcare routines.

PU was also positively correlated with the reverse-scored TA measure. Because TA items were reverse-scored before analysis, this positive correlation should not be interpreted as indicating that greater PU was associated with greater TA. Rather, the positive coefficient indicates that the numerical PU and reverse-scored TA scores tended to vary in the same direction according to the applied scoring procedure. Previous research has highlighted TA as an important factor influencing the acceptance of digital health technologies [69,70,71]. Accordingly, the present association should be interpreted in the context of the scoring procedure and as an interrelationship between TAM-related constructs rather than as evidence that greater PU was accompanied by greater TA.

In contrast, QoL was not significantly associated with the remaining TAM-based domains, and PU was not significantly correlated with overall technology acceptance. These findings suggest that technology acceptance represents a multidimensional construct that cannot be explained solely by general health status or perceived interface usability. Previous studies have similarly reported that psychosocial factors, digital literacy, motivation, perceived health benefits, and previous experience with technology often exert a greater influence on technology acceptance than clinical characteristics alone [21,72].

Overall, our findings indicate that patients with CVDs demonstrated a favorable level of acceptance toward wearable monitoring technology. The observed relationships among PU, SE, and the reverse-scored TA measure further support the relevance of the TAM as a useful framework for understanding patient engagement with wearable health technologies in cardiovascular care [6,38,61,62].

### 4.3. Factors Associated with PEU and Technology Acceptance

The present study demonstrated that demographic and clinical characteristics exerted only a limited influence on participants’ perceptions of the wearable monitoring system. Neither age nor comorbidity burden was significantly associated with PEU, and none of the evaluated participant characteristics independently predicted SUS scores. These findings suggest that the usability of the wearable monitoring system was perceived consistently across individuals with different demographic and clinical profiles, supporting the importance of user-centered device design in facilitating technology use [73,74,75].

The absence of significant predictors of PEU is consistent with previous studies reporting that intuitive interfaces, automated data collection, and simplified interaction reduce the influence of demographic characteristics on usability outcomes [45,51,76]. Modern wearable technologies have increasingly been designed to minimize user effort through passive physiological monitoring, simplified interfaces, and automated synchronization with mobile applications. Consequently, PEU may depend more on the quality of system design than on patient age, educational level, or comorbidity burden [77,78,79].

In contrast, age showed a statistically significant positive regression coefficient in the multivariable analysis, indicating that increasing age was associated with a higher overall TAM-based score and therefore a less favorable technology acceptance profile according to the applied scoring direction. However, because the overall regression model was not statistically significant, this association should be considered exploratory, interpreted cautiously, and confirmed in larger studies. This finding only partially supports our initial hypothesis and suggests that age may influence broader perceptions of technology acceptance rather than the practical usability of the wearable monitoring system itself. Previous studies have reported inconsistent findings regarding the relationship between age and digital health acceptance. While some investigations have shown lower technology acceptance among older adults due to limited digital literacy and previous experience with technology, others have demonstrated that older patients readily adopt wearable technologies when these devices provide clear clinical benefits and adequate user support [52,80,81,82,83].

An important observation was the significant positive correlations among PU, SE, and the reverse-scored TA measure. Given the applied scoring procedure, the positive correlations involving TA reflect covariation with the reverse-scored TA score and should not be interpreted as indicating that greater PU or SE was associated with greater TA. The positive association between PU and SE nevertheless supports the theoretical relevance of users’ perceptions of usefulness and confidence in using technology as important dimensions of technology acceptance [53,84,85]. QoL was not significantly associated with the other TAM-based domain, suggesting that acceptance of wearable monitoring technologies may be more closely related to psychological and behavioral factors than to perceived health-related QoL. Similar conclusions have been reported in studies evaluating digital health interventions among patients with chronic diseases, where motivation, perceived benefit, and digital confidence often outweighed demographic or disease-related characteristics in predicting technology adoption [64,86,87].

Overall, these findings indicate that technology acceptance and PEU represent related but distinct constructs. While usability appears to depend predominantly on the characteristics of the wearable system itself, technology acceptance reflects a broader interaction between users’ perceptions, attitudes, and confidence in digital health technologies. Understanding these differences may facilitate the development of more personalized implementation strategies for wearable monitoring in cardiovascular care [88,89,90,91,92].

### 4.4. Clinical Implications

The findings of the present study have several important implications for the implementation of wearable monitoring technologies in cardiovascular care. As healthcare systems continue to expand the use of telemedicine and remote patient monitoring, patient acceptance and usability have become critical determinants of the long-term success of digital health interventions. The high PEU observed in our study suggests that wearable monitoring systems can be successfully integrated into routine clinical practice, supporting continuous monitoring of patients with chronic CVDs while reducing dependence on traditional face-to-face consultations.

An important clinical implication is that PEU was not significantly influenced by age, comorbidity burden, residential environment, or professional status. This finding suggests that appropriately designed wearable technologies may be feasible across patients with differing demographic and clinical characteristics within the present cohort; however, the study was not powered to establish comparable usability across specific cardiovascular diagnoses. From a clinical perspective, this supports the wider implementation of wearable monitoring systems without restricting their use to younger or technologically experienced populations, provided that adequate patient education and technical support are available.

The observed associations among PU, SE, and technology acceptance further emphasize the importance of patient education when implementing digital health interventions. Previous studies have demonstrated that improving patients’ digital literacy and confidence in using wearable technologies enhances long-term adherence and facilitates successful integration into chronic disease management programs. Consequently, educational interventions, individualized training sessions, and continuous technical support may accompany the introduction of wearable monitoring systems to maximize patient engagement and sustained use.

From a healthcare system perspective, wearable technologies have the potential to support earlier identification of clinical deterioration, facilitate personalized follow-up, and improve communication between patients and healthcare professionals. These advantages may contribute to more efficient outpatient management, reduce unnecessary hospital visits, and optimize healthcare resource utilization, particularly for patients requiring long-term monitoring of chronic cardiovascular conditions. Although the present study did not evaluate clinical outcomes or cost-effectiveness directly, the favorable usability and technology acceptance observed among participants represent important prerequisites for the successful implementation of remote monitoring programs in cardiovascular medicine.

Overall, our findings support the integration of wearable monitoring technologies into routine cardiovascular care as part of patient-centered digital health strategies. However, successful implementation should extend beyond technological innovation and include structured patient education, user-centered system design, and ongoing clinical support to ensure sustained engagement and maximize potential clinical benefits.

### 4.5. Strengths and Limitations

The present study has several strengths. First, it represents one of the few studies to simultaneously evaluate both PEU and technology acceptance of a wearable monitoring system among patients with CVDs using the validated SUS together with an adapted TAM-based questionnaire. Assessing both constructs provided a comprehensive understanding of patients’ experiences with wearable technology, extending beyond simple usability assessment.

Second, the study combined descriptive analyses with correlation and multivariable regression analyses, allowing the exploration of demographic and clinical factors potentially associated with usability and technology acceptance. This analytical approach enabled a more comprehensive evaluation of patient-related associated factors while controlling for potential confounding variables.

Third, the study population consisted of patients with established CVDs who represent a clinically relevant group for the implementation of remote monitoring technologies. As wearable devices are increasingly incorporated into routine cardiovascular care, understanding patients’ perceptions and acceptance is essential for successful clinical implementation and long-term adherence.

Nevertheless, several limitations should be acknowledged. First, although participants used the wearable monitoring system for six months, PEU and technology acceptance were assessed only once, at the end of the monitoring period, and no control group or repeated assessments were included. Consequently, the study cannot determine how usability and technology acceptance evolved over time or exclude potential temporal effects, including an initial novelty effect, habituation, or declining engagement associated with prolonged device use. Repeated assessments at baseline or shortly after device introduction, at an intermediate time point, and at the end of follow-up would have allowed characterization of the trajectory of patient perceptions throughout the monitoring period. In addition, the absence of a control or comparison group limits the ability to distinguish effects related specifically to the wearable monitoring intervention from other time-related influences. Future longitudinal studies incorporating repeated SUS and technology-acceptance assessments and, where appropriate, a comparison group are needed to distinguish initial enthusiasm from sustained acceptance of wearable monitoring technologies.

Second, the study was conducted at a single center and included a relatively small sample of 60 participants. The relatively small sample size limits the statistical precision of the analyses and the generalizability of the findings and may have reduced the ability to detect small associations between participant characteristics and usability or technology acceptance. Moreover, individual cardiovascular diagnostic subgroups were unevenly represented and, in some cases, very small, including valvular heart disease and heart rhythm disorders (*n* = 5, 8.3% each). Consequently, the study was not sufficiently powered for diagnosis-specific subgroup analyses, and the present findings should not be generalized to individual CVD categories. The effect estimates may also have limited precision, as reflected by the CIs, and small or moderate associations cannot be excluded. The regression analyses should therefore be considered exploratory and interpreted cautiously. Larger, adequately powered multicenter studies are needed to confirm the present findings and improve their generalizability.

Third, the study population had a relatively low comorbidity burden, as reflected by a median CCI score of 1.0 (IQR = 1.0). Consequently, the present cohort may not fully represent older, frailer cardiovascular populations or patients with severe multimorbidity, who may experience greater physical, cognitive, or technological barriers to wearable-device use. Therefore, the favorable usability and technology acceptance observed in this study should be generalized cautiously to these more vulnerable patient groups. Future studies should specifically include frailer and more multimorbid cardiovascular populations to determine whether these findings remain applicable across a broader spectrum of clinical complexity.

Fourth, PEU and technology acceptance were assessed using self-reported questionnaires. Although both the SUS and TAM are widely validated instruments, self-reported measures may be influenced by response bias, recall bias, or social desirability bias. Objective indicators of wearable use, such as automatically recorded device adherence, frequency of use, or interaction logs, were not available and could provide complementary information in future investigations.

Finally, the present study focused specifically on patient-reported usability and technology acceptance rather than on the clinical effectiveness of wearable monitoring. Although the wearable device continuously recorded physiological and behavioral parameters during the six-month monitoring period, including daily step count, distance traveled, energy expenditure, heart rate, stress level, and sleep duration, longitudinal changes in these measures were outside the scope of the present analysis. Consequently, the current findings should not be interpreted as evidence regarding the clinical effectiveness of the wearable intervention or its effects on physiological and behavioral outcomes. The study also cannot determine whether the favorable usability and technology acceptance observed translate into improved treatment adherence, better cardiovascular control, reduced hospitalization rates, or improved long-term clinical outcomes. These aspects require dedicated longitudinal analyses and further investigation in prospective studies.

Overall, despite these limitations, the study provides valuable evidence regarding patient acceptance of wearable monitoring technologies in cardiovascular care and contributes to the growing body of literature supporting the implementation of digital health interventions in routine clinical practice.

### 4.6. Future Directions

The findings of the present study provide several directions for future research. First, larger multicenter studies involving more diverse and appropriately stratified cardiovascular populations are needed to validate the present findings and improve their generalizability. Such studies should specifically investigate whether usability and technology acceptance differ across cardiovascular diagnoses, particularly because individual diagnostic subgroups were insufficiently represented in the present cohort for diagnosis-specific analyses. Including patients with different cardiovascular diagnoses, socioeconomic backgrounds, and levels of digital literacy would provide a more comprehensive understanding of the factors influencing wearable technology adoption.

Second, future investigations should adopt longitudinal study designs with repeated assessments of PEU and technology acceptance at baseline or shortly after device introduction, at intermediate time points, and at the end of follow-up. Such repeated measurements would help characterize changes in patient perceptions over time and distinguish an initial novelty effect from habituation or sustained technology acceptance during prolonged device use. Long-term follow-up would also help determine whether sustained engagement with wearable monitoring is associated with clinical outcomes, treatment adherence, and patient satisfaction.

Another important direction involves integrating subjective patient-reported outcomes with objective measures of wearable device utilization. Future studies should combine validated questionnaires, such as the SUS and the TAM, with objective adherence metrics obtained directly from wearable devices, including duration of wear, frequency of use, synchronization rates, and physiological data acquisition. Such an approach would provide a more comprehensive evaluation of patient engagement and enable comparisons between perceived and actual technology use.

Future research should also investigate whether wearable monitoring translates into meaningful clinical benefits for patients with CVDs. Randomized controlled trials and prospective cohort studies should evaluate the effects of wearable technologies on clinical endpoints such as blood pressure control, arrhythmia detection, hospitalization rates, medication adherence, health-related QoL, and cardiovascular mortality. Demonstrating improvements in these outcomes would provide stronger evidence supporting the routine implementation of wearable monitoring within cardiovascular care pathways.

Finally, rapid advances in artificial intelligence, machine learning, and predictive analytics are creating new opportunities to enhance wearable monitoring systems. Future wearable platforms may integrate continuous physiological monitoring with AI-based algorithms capable of identifying early signs of clinical deterioration, personalizing patient feedback, and supporting clinical decision-making. In addition, future studies should evaluate the cost-effectiveness, interoperability, data security, and ethical aspects of AI-assisted wearable monitoring to facilitate its safe and sustainable integration into routine healthcare practice.

Overall, future research should move beyond evaluating patient acceptance alone and focus on demonstrating how wearable monitoring technologies can improve clinical outcomes, optimize healthcare delivery, and support personalized cardiovascular care in real-world clinical settings.

## 5. Conclusions

The present study demonstrated that the evaluated wearable monitoring system achieved high PEU and favorable technology acceptance among patients with CVDs. Most participants rated the system as having good or excellent usability, indicating that wearable technologies may be suitable for integration into routine patient self-monitoring. Although age showed a statistically significant association with technology acceptance, the overall regression model was not statistically significant. Therefore, this observation should be considered exploratory and confirmed in future studies.

The significant associations observed among PU, SE, and technology acceptance emphasize the relationships among cognitive factors associated with acceptance of wearable health technologies. These findings suggest that successful implementation may depend not only on technological performance but also on patients’ confidence in using digital health solutions and their perception of the benefits provided by continuous monitoring.

Overall, this study contributes to the growing evidence supporting the use of wearable monitoring technologies in cardiovascular care. High levels of usability and technology acceptance suggest that wearable devices may represent a feasible and acceptable approach for supporting patient engagement and remote monitoring in cardiovascular care. Future multicenter longitudinal studies incorporating objective measures of device utilization and clinical outcomes are warranted to confirm these findings and further define the role of wearable technologies in routine cardiovascular practice.

## Figures and Tables

**Table 1:** Baseline demographic, clinical, and behavioral characteristics of the study cohort.

Characteristic	Value
Demographic characteristics	
Age, years, mean ± SD	59.0 ± 10.07
Age, years, median (IQR)	59.0 (12.5)
Rural residence, *n* (%)	19 (31.7)
Professionally active, *n* (%)	38 (63.3)
Clinical characteristics	
Hypertension, *n* (%)	49 (81.7)
Heart failure (NYHA class I–II), *n* (%)	7 (11.7)
Valvular heart disease, *n* (%)	5 (8.3)
Heart rhythm disorders, *n* (%)	5 (8.3)
Diabetes mellitus, *n* (%)	8 (13.3)
Other comorbidities *, *n* (%)	54 (90.0)
CCI, mean ± SD	1.0 ± 1.45
CCI, median (IQR)	1.0 (1.0)
Wearable device use	
Continuous device use throughout the study, *n* (%)	40 (66.7)
Intermittent device use (>75% of the study period), *n* (%)	15 (25.0)
Sporadic device use (<75% of the study period), *n* (%)	5 (8.3)
Lifestyle modification attributed to wearable monitoring, *n* (%)	15 (25.0)

* Other comorbidities included dyslipidemia, chronic venous insufficiency, pulmonary disease, and other chronic conditions.

**Table 2:** Qualitative classification of SUS scores.

SUS Score	Usability Rating	Participants, *n* (%)
>80.3	Excellent	35 (58.3)
68.0–80.2	Good	10 (16.7)
51.0–67.9	Acceptable	7 (11.7)
<51.0	Poor	8 (13.3)

**Table 3:** Reliability analysis of the SUS.

Statistic	Value
Cronbach’s α	0.730
McDonald’s ω	0.741
95% CI (Cronbach’s α)	0.614–0.818

**Table 4:** Spearman correlation analysis between participant characteristics and PEU.

Variable	Spearman’s ρ	*p*-Value	FDR-Adjusted *p*-Value
Age	0.106	0.422	0.844
CCI	−0.009	0.946	0.946

Note: FDR-adjusted *p*-values were calculated using the Benjamini–Hochberg procedure.

**Table 5:** Multiple linear regression analysis predicting SUS score.

Predictor	B	95% CI	β	*p*-Value
Age	0.12	−0.18 to 0.41	0.12	0.410
Rural residence	−2.05	−7.20 to 3.11	−0.08	0.530
Professional status	3.11	−2.41 to 8.63	0.15	0.310
CCI	−0.14	−4.36 to 4.09	−0.01	0.956

Note: Model statistics: R^2^ = 0.066, adjusted R^2^ = 0.002, F(4,55) = 0.97, *p* = 0.428.

**Table 6:** Descriptive statistics of TAM-based domain.

Domain	Mean ± SD	Median (IQR)	Shapiro–Wilk *p*-Value
PU	1.50 ± 1.02	1.00 (0.67)	<0.001
SE	1.44 ± 0.91	1.00 (0.50)	<0.001
TA	1.84 ± 1.28	1.00 (1.00)	<0.001
QoL	1.54 ± 0.59	1.40 (0.80)	<0.001
Overall TAM-based score	1.59 ± 0.95	1.33 (0.78)	<0.001

Note: Lower PU, SE, QoL, and overall TAM-based score indicate more favorable responses. TA was reverse-scored before analysis; therefore, its numerical direction should be interpreted according to the scoring procedure.

**Table 7:** Spearman’s rank correlation matrix among TAM-based domain and SUS score.

Variable	Statistic	PU	SE	TA	QoL	SUS
1. PU	Spearman’s ρ	—				
	*p*-value	—				
	FDR-adjusted *p*-value	—				
2. SE	Spearman’s ρ	0.441	—			
	*p*-value	<0.001	—			
	FDR-adjusted *p*-value	<0.001	—			
3. TA	Spearman’s ρ	0.647	0.491	—		
	*p*-value	<0.001	<0.001	—		
	FDR-adjusted *p*-value	<0.001	<0.001	—		
4. QoL	Spearman’s ρ	0.082	0.097	0.153	—	
	*p*-value	0.534	0.461	0.244	—	
	FDR-adjusted *p*-value	0.668	0.668	0.610	—	
5. SUS score	Spearman’s ρ	0.118	0.051	−0.041	0.091	—
	*p*-value	0.372	0.701	0.758	0.489	—
	FDR-adjusted *p*-value	0.668	0.758	0.758	0.668	—

Note: FDR-adjusted *p*-values were calculated using the Benjamini–Hochberg procedure across the 10 pairwise correlations. Correlation coefficients refer to the numerical domain scores according to the scoring procedure described in the Methods. TA was reverse-scored before analysis; therefore, positive correlations involving TA refer to the reverse-scored TA measure and should not be interpreted as positive associations with greater TA.

**Table 8:** Multiple linear regression analysis predicting overall TAM-based score.

Predictor	B	95% CI	β	*p*-Value
Age	0.33	0.02 to 0.64	0.328	0.037
Rural residence	−0.20	−0.58 to 0.18	−0.142	0.291
CCI	−0.06	−0.32 to 0.21	−0.061	0.652

Note: Lower overall TAM-based score indicates more favorable technology acceptance; therefore, positive coefficients indicate movement toward a less favorable acceptance score. Model statistics: R^2^ = 0.109, adjusted R^2^ = 0.061, F(3,56) = 2.28, *p* = 0.088.

## Data Availability

The data presented in this study are available from the corresponding author upon reasonable request. The data are not publicly available due to ethical and privacy restrictions related to participant confidentiality.

## Supplementary Materials

The following supporting information can be downloaded at: https://www.mdpi.com/article/10.3390/medicina62081607/s1, File S1: Supplementary Table S1. Items, construct allocation, scoring, and theoretical sources of the adapted TAM-based questionnaire.

## Abbreviations

The following abbreviations are used in this manuscript:

CCI	Charlson Comorbidity Index
CI	Confidence Interval
CVD	Cardiovascular Disease
DIGI4Care	Digital Medical Technologies Reshaping the Danube Region Healthcare Systems Throughout the Patient Journey from Prevention to Rehabilitation
IQR	Interquartile Range
NYHA	New York Heart Association
QoL	Quality of Life
SD	Standard Deviation
SE	Self-efficacy
SUS	System Usability Scale
TAM	Technology Acceptance Model
TA	Technology Anxiety
PEU	Perceived Ease of Use
PU	Perceived Usefulness
