# Supplementary material for: Usability and Technology Acceptance of a Wearable Monitoring System Among Patients with Cardiovascular Diseases: A Cross-Sectional Study
Source: Medicina (Kaunas). 2026 Aug 21;62(8):1607. doi: 10.3390/medicina62081607 (PMC13515956; doi:10.3390/medicina62081607)
Supplement: Supplementary file 1 [file medicina-62-01607-s001.zip › medicina-4458581-supplementary.pdf]

**File S1: Supplementary Table S1. Items, construct allocation, scoring, and theoretical sources of the adapted TAM-based question-naire**

| Item | Domain | Questionnaire item | Response scale | Scoring/interpretation | Theoretical source |
|---|---|---|---|---|---|
| 1 | Perceived Usefulness (PU) | Using the technology would improve my efficiency in daily activities. | 5-point Likert scale | Lower score = more favorable perception | Davis (1989) [31]; classic TAM—perceived usefulness construct |
| 2 | Perceived Usefulness (PU) | I would find the technology useful in my daily activities. | 5-point Likert scale | Lower score = more favorable perception | Davis (1989) [31]; classic TAM—perceived usefulness construct |
| 3 | Self-efficacy (SE) | I am confident in my ability to use the technology effectively. | 5-point Likert scale | Lower score = more favorable perception | Compeau and Higgins (1995) [32]; technology/computer self-efficacy construct |
| 4 | Self-efficacy (SE) | I could become proficient in using the technology. | 5-point Likert scale | Lower score = more favorable perception | Compeau and Higgins (1995) [32]; technology/computer self-efficacy construct |
| 5 | Self-efficacy (SE) | I could complete a task using the technology if someone showed me how to do it. | 5-point Likert scale | Lower score = more favorable perception | Compeau and Higgins (1995) [32]; technology/computer self-efficacy construct |
| 6 | Technology Anxiety (TA) | I feel apprehensive about using the technology. | 5-point Likert scale | Reverse-scored before calculation of the TA domain and overall TAM-based score | Compeau and Higgins (1995) [32]; technology/computer anxiety-related construct |
| 7 | Technology Anxiety (TA) | I hesitate to use the technology for fear of making mistakes that I cannot correct. | 5-point Likert scale | Reverse-scored before calculation of the TA domain and overall TAM-based score | Compeau and Higgins (1995) [32]; technology/computer anxiety-related construct |

| | | | | | |
|---|---|---|---|---|---|
| 8 | Perceived impact on Quality of Life (QoL) | How would you rate your general health? | 5-point Likert scale | Lower score = more favorable perception | Health-related contextual construct; conceptually informed by WHOQOL-BREF [33] |
| 9 | Perceived impact on Quality of Life (QoL) | How well are you able to concentrate? | 5-point Likert scale | Lower score = more favorable perception | WHOQOL-BREF [33]; psychological domain (concentration) |
| 10 | Perceived impact on Quality of Life (QoL) | How satisfied are you with your personal relationships? | 5-point Likert scale | Lower score = more favorable perception | WHOQOL-BREF [33]; social relationships domain (personal relationships) |
| 11 | Perceived impact on Quality of Life (QoL) | How satisfied are you with the support received from friends and family? | 5-point Likert scale | Lower score = more favorable perception | WHOQOL-BREF [33]; social relationships domain (social support) |
| 12 | Perceived impact on Quality of Life (QoL) | How satisfied are you with your quality of life? | 5-point Likert scale | Lower score = more favorable perception | Overall QoL construct; conceptually informed by WHOQOL-BREF [33] |

Note: Abbreviations: PU, perceived usefulness; SE, self-efficacy; TA, technology anxiety; QoL, quality of life; TAM, Technology Acceptance Model. Scoring note: Domain scores were calculated as the arithmetic mean of the items assigned to each domain. TA items were reverse-scored before calculation of the TA domain and overall TAM-based score. The overall TAM-based score was calculated by averaging the scored questionnaire responses. Lower PU, SE, QoL, and overall scores indicate more favorable perceptions and greater technology acceptance; the TA domain should be interpreted according to its reverse-scored direction.
